# The clinical characteristics and quality of life of 248 pediatric and adult patients with Congenital Adrenal Hyperplasia

**DOI:** 10.3389/fendo.2023.1122435

**Published:** 2023-06-06

**Authors:** Edi A. Shafaay, Mohammed A. Aldriweesh, Ghadeer L. Aljahdali, Amir Babiker, Abdulrahman O. Alomar, Khulood M. Alharbi, Haneen Aldalaan, Ahmed Alenazi, Abdulaziz S. Alangari, Afaf Alsagheir, Bas P. H. Adriaansen, Hedi L. Claahsen – van der Grinten, Ibrahim Al Alwan

**Affiliations:** ^1^ College of Medicine, King Saud Bin Abdulaziz University for Health Sciences, Riyadh, Saudi Arabia; ^2^ King Abdullah International Medical Research Center, Riyadh, Saudi Arabia; ^3^ Department of Pediatrics, King Abdullah Specialized Children’s Hospital, Ministry of National Guard Health Affairs, Riyadh, Saudi Arabia; ^4^ Department of Pediatrics, College of Medicine, Taibah University, Madinah, Saudi Arabia; ^5^ Department of Pediatrics, King Saud Medical City, Riyadh, Saudi Arabia; ^6^ Department of Pediatrics, King Faisal Specialist Hospital and Research Centre, Riyadh, Saudi Arabia; ^7^ Department of Pediatrics, Prince Mohammed Bin Abdulziz Medical City, Aljouf, Saudi Arabia; ^8^ Department of Epidemiology and Biostatistics, College of Public Health and Health Informatics, King Saud bin Abdulaziz University for Health Sciences, Riyadh, Saudi Arabia; ^9^ College of Medicine, Alfaisal University, Riyadh, Saudi Arabia; ^10^ Department of Pediatrics, Radboud Amalia Children’s Hospital, Radboud University Medical Center, Nijmegen, Netherlands

**Keywords:** CAH, psychosocial, QOL, ambiguous genitalia, virilization

## Abstract

**Background:**

Congenital Adrenal Hyperplasia (CAH) is a chronic disease that requires lifelong treatment. Patients may face stigmatization, which may affect their quality of life (QoL). Therefore, we assessed the clinical characteristics and QoL of patients with CAH in the Middle East.

**Methods:**

This case-control study included patients with CAH aged >5 years from two tertiary centers (2020–2021). The patients were matched to a healthy control group and were then divided into pediatric and adult groups. Data were collected from their electronic medical records. Additionally, the EQ-5D-5L QoL questionnaire was completed by both the patients and control group to assess five domains (mobility, self-care, usual activities, pain/discomfort, and anxiety/depression).

**Results:**

The study included 248 patients with CAH (females: 58.8%), with a family history of the condition (57.3%) and/or parental consanguinity (68.1%). The most frequently reported gene defect was CYP21A2, while the most commonly reported symptoms/signs were ambiguous genitalia and obesity. Almost all female patients had received corrective surgery. The questionnaire response rate was 86.3% (n=214/248). The CAH patient group’s mean total QoL score was 85.2 compared with 99.8 in the control. Further, CAH patients had lower QoL scores in all domains compared to those in the control group (p ≤ 0.0001–0.0023). The pain/discomfort and anxiety/depression domains were affected significantly more than the other domains were, with 47.7% and 44.4% participants, respectively, p<0.0001. Additionally, obesity was found to be a predictor of reduced mobility following a logistic regression analysis (p ≤ 0.04, OR (0.18-0.98)).

**Conclusion:**

Patients with CAH reported lower QoL overall, particularly in the pain/discomfort and anxiety/depression domains. Based on this, we recommend the early involvement of psychologists in a multidisciplinary team approach, pre-marital screening, and the implementation of awareness programs for people diagnosed with CAH in communities with high consanguineous mating.

## 1 Introduction

Congenital Adrenal Hyperplasia (CAH) is an autosomal recessive disease caused by mutations or deletions among genes that encode enzymes producing sex steroids, mineralocorticoids, and glucocorticoids in the adrenal glands. Herein, 21-hydroxylase enzymes are the most commonly affected, resulting in impaired production of cortisol, mineralocorticoids, and elevated androgens and leading to virilization of varying degrees at birth in female patients (1). CAH is a chronic illness with a spectrum of clinical, biochemical, and genetic presentations that are likely to affect patients’ quality of life (QoL) (2). Moreover, in some countries, patients with CAH suffer from a lack of access to affordable medical care and knowledge about their medical condition, societal stigmatization, and negative social beliefs related to intersex, cultural, and religious issues (2). For example, cultural factors, such as male predominance in certain societies, may influence peoples’ decisions regarding gender assignment and reassignment, which may impact the QoL of patients with CAH throughout their life (2, 3). CAH patients’ QoL can also be affected by their clinical features; for example, the recurrent presentation of severe vomiting, dehydration, and shock along with hypertension has been observed in patients with 11-hydroxylase deficiency (4). Excess androgens may also lead to excessive facial hair and early penile enlargement in males. Moreover, untreated female patients can experience excessive facial and body hair, a relatively deep voice, anovulation, and menstrual irregularities (4). In addition, both sexes can develop precocious puberty and a reduced final height due to rapid growth and early closure of the growth plate, in addition to puberty failure (1, 4–6). Herein, adjusting glucocorticoid doses is crucial to avoid overtreatment, which can lead to cushingoid habitus and undertreatment with the above features that further affect QoL in pediatric and adult patients (1, 5).

“QoL is defined as “individual's perception of their position in life in the context of the culture and value systems in which they live and in relation to their goals, expectations, standards and concerns” (7). Unfortunately, CAH patients often suffer from several physical and psychological complications despite early medical care. For instance, growth failure, obesity, infertility, adrenal masses, hypertension, cataract, and emotional and psychosocial sequelae may negatively affect their well-being and QoL. The extant literature indicates a discrepancy in QoL levels in these patients. However, there is lack of reports focusing on QoL in CAH patients in the Middle East (8–13). Additionally, most patients in the Middle East are affected by various social and religious factors, which require special consideration in the treatment of patients with CAH (4). A patient’s cultural values and religious obligations can affect their decision-making in different aspects of CAH management. Furthermore, because of the social advantages associated with male assignments in Arab societies and other similar communities, early assignment with the male sex is likely to be valued more in cases of ambiguity.

Therefore, this study aimed to assess the QoL and clinical characteristics of pediatric and adult Saudi patients with CAH.

## 2 Materials and methods

### 2.1 Study design and setting

This retrospective case-control study was conducted at King Abdullah Specialized Children’s Hospital (KASCH) and King Faisal Specialist Hospital and Research Centre (KFSH&RC) in Riyadh, Saudi Arabia.

### 2.2 Study participants

We included patients aged >5 years who were diagnosed with classic CAH from both KASCH and KFSH&RC from July 2020 to July 2021. The study sample comprised two groups: patients with CAH and matched healthy controls. For the CAH group, we included all pediatric and adult patients with a CAH diagnosis confirmed by clinical, genetic, and laboratory tests. In addition, the criteria for the control group consisted of 1) healthy siblings of patients with CAH, 2) with the nearest age, 3) and same-sex, as applicable. For CAH patients without siblings of the same sex, we enrolled siblings of the opposite sex. For patients without siblings, the sibling of another patient with CAH was enrolled considering the nearest age.

### 2.3 Questionnaire

A qualitative interview was conducted with all participants to deliver the questionnaire and discuss the impact of and concerns regarding CAH. Specifically, we used the EQ-5D-5L questionnaire to measure QoL in our study sample. This questionnaire consists of five domains (mobility, self-care, usual activities, pain/discomfort, and anxiety/depression) and uses a scale of 0–100 to represent a respondent’s overall health status (14). A 5-point Likert scale is used for each domain. The total scores are positively proportional, meaning that higher scores indicate higher QoL. The EQ-5D-5L questionnaire was translated and validated in numerous languages by the EuroQol group. Further, permission was obtained from the EuroQol to use the Arabic version in this study. Additionally, the EQ-5D-5L questionnaire was validated in several studies for use among people with chronic diseases, such as asthma, diabetes mellitus, and cardiovascular diseases, and showed good validity and reliability (15–17).

### 2.4 Data collection

Owing to the COVID-19 pandemic, questionnaire responses were collected through telephonic interviews. Furthermore, informed consent was obtained from each participant. For those aged under 18 years, assent was taken from the child, with consent obtained from their parents. The patients completed the questionnaire independently, other than young children, who completed them with the assistance of their legal guardians.

All medical records were reviewed after obtaining consent via telephone. The collected data included patients’ demographic details, including age, sex, sex of rearing, and clinical data (height, weight, body mass index (BMI) type of CAH, gene defect, date of diagnosis, presence of ambiguous genitalia, corrective surgery experienced, comorbidities, complications, parental consanguinity, family history of CAH, and medications used). In addition, these patients received medical follow-up from the day of diagnosis until their current age.

### 2.5 Statistical analysis

The descriptive variables are presented as percentages and frequencies based on the data distribution of the means (± SD) or medians (interquartile range). Inferential analyses were used to compare the pediatric and adult groups in terms of the questionnaire domains using a t-test for numerical data and a chi-square test for categorical data. Logistic regression analysis was performed to assess the predictors of reduced QoL among the various CAH complications. For the final inferences, results with p- values of less than 0.05 with two-sided testing were considered significant.

### 2.6 Ethical approval

The study was approved by the Institutional Review Board (IRB) of the King Abdullah International Medical Research Center (KAIMRC) and the KFSH&RC, Riyadh, Kingdom of Saudi Arabia.

## 3 Results

### 3.1 Description of the final study sample

The final sample comprised 248 eligible participants. The demographics and clinical characteristics of the pediatric group are shown in **Table 1**. The majority (58.8%, n=146/248) were in the pediatric group (mean age 11.5 ± 4.0 years for the males, 11.7 ± 3.9 years for females). Two pediatric males (46XY) were raised as females; both had a StAR gene deficiency. Additionally, CYP21 was the most frequently affected gene in the pediatric group (89%, n=130/146), followed by CYP11 (4.8%, n=7/146). The presence of ambiguous genitalia was the most common presentation (59.6%, n=87/146), followed by obesity (31.5%, n=46/146) and testicular adrenal rest tumors (TARTs) among 10 patients. Furthermore, parental consanguinity and a family history of CAH were reported in 65.8% and 56.8% of the patients, respectively.

**Table 1:** The demographic and clinical characteristics of the pediatric group aged 6–17 years with Congenital Adrenal Hyperplasia (CAH) (N = 146).

	Male (N = 57)	Female (N = 89)
**Mean Age (years)** (SD)	11.5 (4)	11.7 (3.9)
**Opposite sex of rearing**	0 (0)	2 (1.4)
Gene defect		
CYP21	50 (87.7)	80 (89.9)
CYP11	1(1.8)	6 (6.7)
StAR	2 (3.5)	2 (2.2)
HSD3	3 (5.3)	0 (0.0)
CYP17	1 (1.8)	0 (0.0)
StAR/ACY11A1	0	1 (1.1)
Medical and Surgical history		
Ambiguous Genitalia	4 (7.01)	83 (93.3)
Obesity	17 (29.8)	29 (32.6)
Precocious Puberty	15 (26.3)	19 (21.3)
Adrenal Crisis	5 (8.8)	9 (10.1)
Incorrect sex at birth	2 (3.5)	11 (12.4)
Testicular Adrenal rest tumor	10 (17.5)	0 (0.0)
Hyperglycemia	2 (3.5)	0 (0.0)
Primary Gonadal Failure	0 (0.0)	1 (1.1)
Corrective surgery	7 (12.3)	82 (92.1)
Two or more corrective surgeries	1 (1.8)	6 (6.7)
Any Surgery Complication	1 (1.8)	10 (11.2)
**Parents Consanguinity**	34 (59.6)	62 (69.7)
**Family History of CAH**	30 (52.6)	53 (59.6)

The clinical characteristics of the adult patients with CAH are shown in **Table 2**. They constituted 41.1% (n=102) of the sample, with the majority (55.9%, n=57/102) being female (mean age for males was 25.6 ± 6.4 years and was 26 ± 6.6 years for females). A male sex was assigned to two adult females (46XX) with CYP21 and CYP11 gene mutations. The CYP21 gene defect was the most frequently observed (91.2%, n=93/102), followed by defects in CYP11 (4.9%, n=5/102) and HSD3 (3.9%, n=4/102). Among the reported CAH complications, a short stature was the most prominent (67.7%, n=69/102), with ambiguous genitalia being present in 52.9% (n=54/102), followed by obesity in 39.2% (n=40/102). TARTs were detected in 12 patients. Parental consanguinity and a family history of CAH were reported in 71.6% and 57.8% of patients, respectively.

**Table 2:** The demographic and clinical characteristics of the adult group aged 18–45 with Congenital Adrenal Hyperplasia (CAH) (N = 102).

	Male (N = 45)	Female (N = 57)
**Mean age (years) (SD)**	25.6 (6.4)	26.0 (6.6)
**Opposite sex of rearing**	2 (2)	0 (0.0)
Body measurement		
Mean Height (cm) (SD)	156.8 (11.1)	147.3 (14.4)
Mean Weight (kg) (SD)	69.3 (19.2)	61.7 (19.3)
Mean (BMI) (kg/m2) (SD)	28.6 (7.5)	28.2 (7.2)
Gene defect		
CYP21	40 (88.9)	53 (93.0)
CYP11	3 (6.7)	2 (3.5)
HSD3	2 (4.4)	2 (3.5)
Medical and Surgical history		
Ambiguous Genitalia	2 (4.4)	52 (91.2)
Obesity	14 (31.1)	26 (45.6)
Short Stature	27 (60.0)	42 (73.7)
Precocious Puberty	6 (13.3)	5 (8.8)
Adrenal Crisis	4 (8.9)	2 (3.5)
Incorrect sex at birth	2 (4.4)	2 (3.5)
Testicular Adrenal rest tumor	12 (26.7)	0 (0.0)
Oligomenorrhea	0 (0.0)	9 (15.8)
Primary Amenorrhea	0 (0.0)	12 (21.1)
Hyperglycemia	4 (8.9)	4 (7.0)
Primary Gonadal Failure	3 (6.7)	2 (3.5)
Corrective surgery	6 (13.3)	52 (91.2)
Two or more corrective surgeries	0 (0.0)	4 (7.0)
Any Surgery Complication	1 (2.2)	3(5.3)
**Parents Consanguinity**	33 (73.3)	40 (70.2)
**Family History of CAH**	24 (61.4)	35 (61.4)

### 3.2 QoL assessment

The response rate to the QoL questionnaire in CAH patients was 86.3% (n=214/248), and it was 100% (n=214/214) in the control group. The distribution of the questionnaire in adult CAH patients and the control group is shown in **Table 3**. Regarding the “mobility” domain, 12.3% (N=10/81) of adult CAH patients complained of various problems. For the “self-care” domain, only 6.2% (N=5/81) complained that they were affected, while the “usual activities” domain affected 16% (N=13/81) of the participants. The majority of adult patients with CAH (53.1%; N=43/81) were affected at variable degrees in the “pain/discomfort” domain, as well as in the “depression/anxiety” domain, which represents 46.9% (N=38/81) of the adult patients. The median total health score (0-100) was 90, with an IQR of 15. QoL was significantly different in adult patients with CAH in the five domains compared to the healthy control group. We encountered 21 adult CAH patients without siblings of the same sex. Thus, we enrolled controls of the opposite sex (20.6%; adult control group) (**Table 3**).

**Table 3:** Comparison between the adult CAH and control groups’ responses to the EQ-5D questionnaire.

Variable		Adult CAH N=81	Control N=102	*P*
**Mean age (years) (SD)**		26 (6.6)	25.5 (5.6)	0.3
**Gender, n (%)**	Male	36 (44.4)	52 (51)	0.1
	Female	45 (55.5)	50 (49)	
**Mobility, n (%)**	No problem	71 (87.7)	102 (100)	***<.0001***
	Slight problem	6 (7.4)	0 (0)	
	Moderate problem	4 (4.9)	0 (0)	
	Severe problem	0 (0)	0 (0)	
	Unable/Extremely	0 (0)	0 (0)	
**Self-care, n (%)**	No problem	76 (93.8)	214 (100)	***<.015***
	Slight problem	2 (2.6)	0 (0)	
	Moderate problem	1 (1.2)	0 (0)	
	Severe problem	1 (1.2)	0 (0)	
	Unable/Extremely	1 (1.2)	0 (0)	
**Usual Activities, n (%)**	No problem	68 (84)	101 (99)	***<.0021***
	Slight problem	8 (9.9)	1 (1)	
	Moderate problem	4 (4.9)	0 (0)	
	Severe problem	0 (0)	0 (0)	
	Unable/Extremely	1 (1.2)	0 (0)	
**Pain/discomfort, n (%)**	No problem	38 (46.9)	102 (100)	***<.0001***
	Slight problem	19 (23.5)	0 (0)	
	Moderate problem	21 (25.9)	0 (0)	
	Severe problem	3 (3.7)	0 (0)	
	Unable/Extremely	0 (0)	0 (0)	
**Depression/anxiety, n (%)**	No problem	43 (53.1)	97 (95.1)	***<.0001***
	Slight problem	12 (14.8)	4 (3.9)	
	Moderate problem	17 (21)	0 (0)	
	Severe problem	6 (7.4)	0 (0)	
	Unable/Extremely	3 (3.7)	1 (1)	
**Median total health scale 0-100 (IQR)**		90 (15)	100 (0)	***<.0001***

The bold values denote statistically significant P values.

Similarly, 15.8% (N=21/133) of pediatric CAH patients reported problems in the “mobility” domain. For the “self-care” domain, only 8.3% (N=11/133) reported any negative experiences. Regarding the “usual activities” domain, 18.8% (N=25/133) reported experiencing various problems. For the “pain/discomfort” domain, 44.5% of patients (N=59/133), which includes the majority of pediatric CAH patients, indicated that they had experienced problems at varying degrees, similar to the “depression/anxiety” domain (42.9%; N=57/133). The median total health score (0–100) was 94, with an IQR of 20 in pediatric patients with CAH. The overall QoL in pediatric patients with CAH was found to be more significantly affected in the five domains compared to that in the healthy control group (**Table 4**). We encountered 63 pediatric CAH patients without siblings of the same sex. Hence, we enrolled controls of the opposite sex (56.3%; pediatric control group) (**Table 4**).

**Table 4:** Comparison between the pediatric CAH and control groups’ responses to the EQ-5D questionnaire.

Variable		Pediatric CAH N=133	Control N=112	*P*
**Mean age (years) (SD)**		11.7 (3.9)	12 (4)	0.5
**Gender, n (%)**	Male	47 (35.3)	68 (60.7)	***<.0001***
	Female	86 (64.6)	44 (39.3)	
**Mobility, n (%)**	No problem	112 (84.2)	112 (100)	***<.0001***
	Slight problem	10 (7.5)	0 (0)	
	Moderate problem	8 (6)	0 (0)	
	Severe problem	1 (0.8)	0 (0)	
	Unable/Extremely	2 (1.5)	0 (0)	
**Self-care, n (%)**	No problem	122 (91.7)	112 (100)	***<.007***
	Slight problem	7 (5.3)	0 (0)	
	Moderate problem	0 (0)	0 (0)	
	Severe problem	2 (1.5)	0 (0)	
	Unable/Extremely	2 (1.5)	0 (0)	
**Usual Activities, n (%)**	No problem	108 (81.2)	112 (100)	***<.0001***
	Slight problem	10 (7.5)	0 (0)	
	Moderate problem	13 (9.7)	0 (0)	
	Severe problem	1 (0.8)	0 (0)	
	Unable/Extremely	1 (0.8)	0 (0)	
**Pain/discomfort, n (%)**	No problem	74 (55.6)	112 (100)	***<.0001***
	Slight problem	28 (21.1)	0 (0)	
	Moderate problem	21 (15.8)	0 (0)	
	Severe problem	9 (6.8)	0 (0)	
	Unable/Extremely	1 (0.8)	0 (0)	
**Depression/anxiety, n (%)**	No problem	76 (57.1)	112 (100)	***<.0001***
	Slight problem	26 (19.6)	0 (0)	
	Moderate problem	23 (17.3)	0 (0)	
	Severe problem	6 (4.5)	0 (0)	
	Unable/Extremely	2 (1.5)	0 (0)	
**Median total health scale 0-100 (IQR)**		94 (20)	100 (0)	***<.0001***

The bold values denote statistically significant P values.

During the qualitative interviews, patients were asked about the factors that affect their QoL in terms of CAH. The answers mainly concerned the lifelong course of the disease, its medications, and the related long-term hospital visits. Some participants reported about CAH affecting their sexual life, either physically or psychologically, and expressed the fear of having affected children of their own. Other answers were those describing disease complications, such as short stature.

### 3.3 Predictors of QoL

Logistic regression analysis results in **Table 5** show that obesity was significantly associated with impaired mobility in our patient groups (OR=0.4 (CI=0.2-1.0); p = 0.04), while precocious puberty was associated with pain/discomfort (OR=0.5 (CI=0.2-1.0); p = 0.04).

**Table 5:** Logistic regression with collapsed variables among adults and pediatrics.

Variables		Age groups (Adults vs. Pediatrics)	BMI (Obese vs. Non-obese)	Ambiguous Genitalia	Adrenal Crisis	Precocious Puberty	Short Stature
**Mobility**	**Unadjusted Odds Ratio**	1.4	0.4	0.9	0.7	1.1	0.6
	**(95% CI)**	(0.6 – 3.1)	***(0.18 – 0.98)***	(0.4 – 1.9)	(0.1 – 3.3)	(0.4 – 2.9)	(0.3 – 1.5)
	***X^2^***	0.6	***4.1***	0.08	0.2	0.03	1.1
	***P***	0.4	***0.04***	0.8	0.7	0.9	0.3
**Self-care**	**Unadjusted Odds Ratio**	1.4	0.6	1.8	0.7	NA	0.7
	**(95% CI)**	(0.5 – 4.3)	(0.2 – 1.8)	(0.5 – 5.7)	(0.1 – 5.7)	NA	(0.2 – 2.2)
	***X^2^***	0.4	0.9	0.9	0.1	NA	0.3
	***P***	0.5	0.3	0.3	0.7	NA	0.6
**Usual Activities**	**Unadjusted Odds Ratio**	1.1	0.7	1.5	1.4	0.5	0.8
	**(95% CI)**	(0.5 – 2.3)	(0.4 – 1.5)	(0.7 – 3.2)	(0.4 – 4.4)	(0.2 – 1.4)	(0.4 – 1.8)
	***X^2^***	0.1	0.6	1.1	0.3	1.8	0.2
	***P***	0.8	0.4	0.3	0.6	0.2	0.7
**Pain/Discomfort**	**Unadjusted Odds Ratio**	0.6	0.7	1.01	1.8	0.5	1.4
	**(95% CI)**	(0.4 – 1.1)	(0.4 – 1.2)	(0.6 – 1.8)	(0.7 – 4.9)	(0.2 – 1.0)	(0.8 – 2.4)
	***X^2^***	2.3	1.5	0.003	1.4	3.9	1.2
	***P***	0.1	0.2	0.9	0.2	0.04	0.3
**Depression/Anxiety**	**Unadjusted Odds Ratio**	0.9	0.8	0.7	1.6	0.9	0.9
	**(95% CI)**	(0.5 – 1.6)	(0.5 – 1.4)	(0.4 – 1.2)	(0.6 – 4.3)	(0.5 – 1.9)	(0.5 – 1.7)
	***X^2^***	0.1	0.4	1.6	0.9	0.01	0.02
	***P***	0.7	0.5	0.2	0.3	0.9	0.9

The bold values denote statistically significant P values.

## 4 Discussion

In our study, the 21-hydroxylase deficiency was the most common gene defect found among CAH patients, followed by CYP11 and HSD3, which is consistent with previous literature (18). Most CAH cases are associated with parents’ consanguinity and a family history of CAH. The most common complications observed in our patients were ambiguous genitalia in neonates, precocious puberty in childhood, and obesity and short stature in adulthood. Almost all female patients in our cohort had received corrective surgery. Further, patients with CAH reported lower QoL in all domains; however, our sample primarily experienced problems in the pain/discomfort and depression/anxiety domains. Moreover, patients reported a lower total health score than the healthy matched controls.

Globally, some studies have assessed QoL in pediatric and adult patients with CAH. Similar to our findings, these studies found CAH patients to have an impaired QoL in all domains compared to healthy participants (9, 19, 20). In the extant literature, QoL was either more significantly compromised in males or females or was equally compromised in both (13, 21–23). Some studies have found no effect of CAH on the QoL of adult patients compared to healthy participants, although males with CAH were found to be less sexually active (24, 25). In the Middle East, specifically, studies assessing QoL in patients with CAH are scant. One study in Egypt assessed QoL in children and adolescents with CAH without using a control group reported an overall reduction in QoL, similar to our study (10). Another Israeli study reported that CAH has no effect on health-related QoL. However, this could be explained by their cohort being clustered in non-classical CAH, which has a favorable disease course (26).

Regarding the QoL domains in our study, many patients had lower scores in the pain/discomfort domain. These complaints were mainly related to increased pain in the limbs, such as that caused by obesity, which significantly limits mobility. The association between precocious puberty and the pain/discomfort domain of QoL remains unexplored in the literature, and we could not find an explanation for this phenomenon.

For the psychological domain assessed in our study, many participants reported being affected by several factors, including the chronicity of the disease, need for lifelong medication, impacts on their sexual life, fear of having affected children, need for continuous hospital visits, and various disease complications, such as short stature. The literature also reports that the psychological domain is among the most concerning in this population (9, 10, 12, 13, 27). Other studies have found increased psychiatric symptoms and morbidity rates in children, adolescents, and adult patients with CAH (19, 27), as well as increased anxiety and suicidal thoughts (12). This is supported by a Swedish study that reported fewer chances of marriage, a lack of sexual activity, and delays in psychosexual development in this population (28). In particular, males with CAH have fewer chances of having a lifetime partner and often experience erectile dysfunction, decreased sexual activity, and reduced fertility, which leads to substantial psychological issues (25, 27). A study on sexual orientation found that females with CAH had increased rates of homosexuality and bisexuality than the control group, which might be explained by excess prenatal androgen exposure (29).

A systematic review concluded that adult CAH males experienced more psychological issues than their female counterparts (11). In contrast, other studies reported that CAH did not significantly affect adult QoL compared to the healthy population, except that CAH patients were less sexually active (24, 25). Another study reported that males with CAH were satisfied with their sexual life with reasonable hormonal control, with the results being comparable to those of a healthy control population (30). Moreover, the frequency of sexual intercourse in patients with CAH did not differ from that in healthy control groups in another study (24). In our study, patients experienced psychosocial challenges similar to those mentioned in previous research. With some limitations to open discussions about psychosexual issues in our communities, most of these challenges should preferably be explored regularly in the routine follow-up of patients with CAH in the Middle East.

Our study has some limitations. First, due to the COVID-19 pandemic, we had to conduct phone interviews, which may have affected our response rate. Second, this was a retrospective study, meaning that we may potentially have missed some documentation in the clinical characteristics section. Third, the questionnaire was a generic tool rather than a disease-specific measure, which could have missed various patients’ concerns and unique issues, such as those concerning their sexuality and the effects of genital surgeries.

## 5 Conclusion

In this study, participants with CAH reported a significantly reduced QoL in all domains, particularly in the pain/discomfort and anxiety/depression domains, as well as lower total health scores compared to healthy controls. As such, we recommend the early involvement of a psychologist in a multidisciplinary team approach for treating CAH patients to improve their mental health outcomes. Further, in areas with a high prevalence of parental consanguinity, testing for CAH during pre-marital screening and awareness programs about genetic diseases such as CAH should be implemented nationwide.

## Data availability statement

The original contributions presented in the study are included in the article/supplementary materials, further inquiries can be directed to the corresponding author.

## Author contributions

ES, MA, GA, IA, AB, AOA, and AfA contributed to the design and implementation of the study and writing of the manuscript. MA, ES, and ASA analyzed the data and wrote the methodology. KA, HA, AhA, AOA, ES, GA, and MA collected data and co-wrote the manuscript. IA, ES, MA, AB, AfA, HC-G, and BA reviewed and edited the final manuscript. IA, AB, and AfA supervised the project. All authors contributed to the article and approved the submitted version.
